# Determining the amount of training needed for competency of anesthesia trainees in ultrasonographic identification of the cricothyroid membrane

**DOI:** 10.1186/s12871-017-0366-7

**Published:** 2017-06-02

**Authors:** Katia F. Oliveira, Cristian Arzola, Xiang Y. Ye, Jefferson Clivatti, Naveed Siddiqui, Kong E. You-Ten

**Affiliations:** 1 Department of Anesthesia and Pain Management, Mount Sinai Hospital-Mount Sinai Health System, 600 University Avenue, Toronto, ON M5G 1X5 Canada; 2 Micare Research Centre, Mount Sinai Hospital-Sinai Health System, 700 University Ave, Toronto, ON M5G 1X6 Canada; 3 Department of Anesthesia and Pain Management, Mount Sinai Hospital-Sinai Health System, University of Toronto, 600 University Avenue, Rm 19-104, Toronto, ON M5G 1X5 Canada

**Keywords:** Competence, Cumulative sum method, Ultrasound, Cricothyroid membrane, Airway management

## Abstract

**Background:**

Airway guidelines recommend the use of ultrasound to localize the cricothyroid membrane prior to airway manipulation in difficult airways. In this study, we aimed to determine the amount of training anesthesia trainees would need to achieve competence in bedside ultrasound to identify the cricothyroid membrane.

**Methods:**

This is a prospective non-randomized cohort study in the Department of Anesthesia at Mount Sinai Hospital (Toronto, Ontario, Canada). Following institutional ethics approval, six anesthesia trainees consisting of four residents and two fellows underwent a 2-h training session on neck ultrasound to identify neck landmarks and the cricothyroid membrane. The trainees had no previous airway ultrasound experience. One-two weeks later, each trainee performed consecutive neck ultrasound scans on 20 healthy volunteers to identify the cricothyroid membrane. Cumulative sum (CUSUM) learning curves were constructed for each trainee. Primary outcome was the number of ultrasound examinations required to achieve competence, defined as 90% success rate in a series of 20 ultrasound scans. Secondary outcomes were the overall success rate, the time (sec.) required to perform the task, and 3-month skills assessment.

**Results:**

CUSUM analysis showed four trainees achieved competence with a mean [range] success rate of 94.0% [90–100%] and a median [range] number of attempts of 14 [9–18]. Two trainees did not achieve competence, but obtained a success rate of 75.0 and 80.0% each. Overall (number of attempts) success rate was 88.3% (106/120) with a mean (SD) time of 36.9 (9.0) sec. Three months after training, ultrasound of five consecutive neck scans showed a mean success rate of 86.7% (26/30) and mean (SD) time of 47.7 (16.0) sec.

**Conclusions:**

After a short 2-h training session, most anesthesia trainees (*n* = 4/6) achieved competence in ultrasound-identification of the cricothyroid membrane with less than 20 scans in a mean time less than 60 s., and that they remain reasonably competent 3 months later. The learning curve for ultrasound identification of the cricothyroid membrane seems to be short even without prior airway ultrasound experience.

## Background

Airway management is an important skill that anesthesiologists must become proficient in. In the worst clinical scenario of a ‘can’t intubate-can’t oxygenate’ airway crisis the anesthesiologist must perform a cricothyrotomy, an emergency procedure that is potentially life-saving [1, 2]. Success depends on understanding the correct neck landmarks [3, 4]. However, misidentification of the cricothyroid membrane is a major cause of tube misplacements leading to cricothyrotomy failures and serious complications [5, 6]. Evidence showed that palpation of the cricothyroid membrane by anesthesiologists is inaccurate, even under elective conditions [3, 7–9]. When ultrasound identification of the cricothyroid membrane is compared to identification by palpation, the imaging modality provides superior success rate and decreased complications [10, 11].

Several airway guidelines for difficult airway management recommend ultrasound to be used to identify neck landmarks and the cricothyroid membrane before airway management in non-emergency situation [12, 13]. The Canadian Society of Anesthesiologists’ guidelines for difficult airway management mention ultrasound as a potentially useful tool to identify the cricothyroid membrane prior to a surgical airway, but outline the need of an experienced operator [12]. Moreover, the Difficult Airway Society guidelines recommend the identification of the cricothyroid membrane during the preoperative assessment and if this is not possible with palpation, ultrasonography should be used [13]. These guidelines recognize that airway evaluation using ultrasound is an invaluable skill for anesthesiologists and training in its use is recommended. Currently, the amount of training required to achieve competence in ultrasound-guided identification of the cricothyroid membrane is not known.

The aim of this study was to determine the amount of training an anesthesiologist would need to become competent in identifying the cricothyroid membrane using ultrasonography in a non-emergency situation.

## Methods

### Study design

The Mount Sinai Hospital Research Ethics Board (Toronto, Ontario, Canada) approved the study and written informed consent was obtained from all anesthesia trainees and volunteers. This was a prospective cohort study of a group of anesthesia trainees learning ultrasound identification of the cricothyroid membrane on healthy volunteers. The trainees were recruited from Mount Sinai Hospital during their clinical anesthesia rotation and were in postgraduate year 1 (*n* = 2), postgraduate year 2 (*n* = 2) and fellows (*n* = 2). Trainees had previous experience in ultrasound-assisted procedures, but not in airway ultrasound and identification of the cricothyroid membrane. Volunteers were males and females of age 18 years and older, without a previous history of neck surgery, neck irradiation and known neck deformity or abnormality. Consented volunteers were submitted to a morphometric assessment of the neck including inspection and palpation of the anatomical neck landmarks, cricothyroid membrane and measurement of neck circumference. The volunteers’ demographics (age, weight, height) were also recorded. Volunteers with visually easy identification of neck landmarks were excluded from the study. The degree of difficulty in identifying the landmarks was graded according to a previously described grading system. [10, 11] Easy = visual landmarks; Moderate = requires light palpation of landmarks; Difficult = requires deep palpation of landmarks; Impossible = landmarks not palpable.

### Ultrasound training

All trainees received 2 h of training consisting of (i) didactic teaching on neck ultrasound-identification of the cricothyroid membrane in the form of reading material [14], an educational video (15 mins) and a Powerpoint® presentation, and (ii) an interactive session that included a live demonstration and hands-on practice with one-on-one feedback by one of the investigators (KEYT). Ultrasonography of the neck was performed using the longitudinal approach [9, 14–16]. A portable ultrasound system equipped with a linear high-frequency (Zonare Medical Systems, Inc., Mountain View, CA, USA) was used to scan the neck. Volunteers were positioned supine, with the neck in a neutral position for examination. The participant stood on the volunteer’s right side facing his/her head. The transducer was held in the dominant hand and placed transversely over the neck just above the suprasternal notch to visualise the trachea. The transducer was then moved laterally to the subject’s right side until the right border of the transducer was superficial to the midline of the trachea. The right end of the transducer was kept in the tracheal midline, while the left end of the transducer was rotated into the sagittal plane to produce a longitudinal scan of the tracheal midline. The transducer was then moved cranially to visualise sequentially the tracheal rings, the cricoid cartilage, cricothyroid membrane and the thyroid cartilage.

### Assessment process

Within 1 week of training, each trainee was assessed to identify the cricothyroid membrane using ultrasonography and to identify the point of entry of a cricothyrotomy tube. The assessment was performed on volunteers positioned supine with the neck in neutral position. Correct ultrasonographic identification of the cricothyroid membrane and point of entry was confirmed by one of the study investigators (KEYT, CA, NS) with at least 6 years of experience in airway ultrasound. Each trainee was asked to point “here” on the ultrasound monitor when the cricothyroid membrane was identified. A series of neck ultrasound on 20 different volunteers consecutively was performed by each trainee, allowing for the construction of individual learning curves through CUSUM [17–19]. Three months after training, the trainees were re-assessed in identifying the cricothyroid membrane of five different healthy adult volunteers using ultrasonography.

The primary outcome was the number of ultrasound scans required to achieve competence in the ultrasound-guided identification of the cricothyroid membrane. Competence was defined as 90% success rate in a series of ultrasound scans [20, 21]. The secondary outcomes included (a) the overall success rate, (b) the time (sec.) required to perform the task, defined as from the time the linear probe was placed transversely above the sternal notch to the identification of the cricothyroid membrane, (c) 3-month skill assessment for correct identification of the cricothyroid membrane using ultrasound and the time (sec.) required to perform the task.

### Statistical analysis

In order to determine the primary outcome for each anesthesiologist, we constructed individual learning curves using the CUSUM graphical method (Appendix). The CUSUM graphs display cumulative differences plotted in sequence, allowing detection of deviations from a predetermined standard in a series of consecutive procedures [17, 18, 22]. If the task is performed successfully the CUSUM Score line progresses downwards, whereas a failure is indicated as an upward trend on the graph. The participant was considered competent reaching up to 90% success rate, if the CUSUM Score reached two decision lines downwards.

Based on previous ultrasound studies [20, 23], we calculated a minimum sample size of 13–18 consecutive neck ultrasound assessments as required to determine competence at an acceptable failure rate of 10% and an unacceptable failure rate of 30%, respectively. Each trainee performed a total of 20 consecutive neck ultrasound assessments to generate the CUSUM graph.

Descriptive statistical methods were used to describe the study population. The statistical analyses were performed using STATA® for Macintosh, Release 13.1 (StataCorp, College Station, TX, USA), SAS® 9.2 (SAS Institute Inc., Cary, NC, USA) and R 10.2 (http://www.r-project.org/). A two-sided significance level of <0.05 was used without multiple comparison adjustment.

## Results

A group of six anesthesia trainees completed the study, consisting of two anesthesia fellows, 2nd-year anaesthesia trainees and two first-year anaesthesia trainees. None of the trainees had previous experience in airway ultrasound. The group of volunteers consisted of 13 females and seven males, with a mean (SD) age of 41 (10) years, weight of 71 (14) kilograms, BMI 25 (4) kgm^−2^ and neck circumference 35 (3.6) cm. The degree of neck landmarks difficulty of the volunteers was graded to be moderate to difficult.

**Fig. 1:**
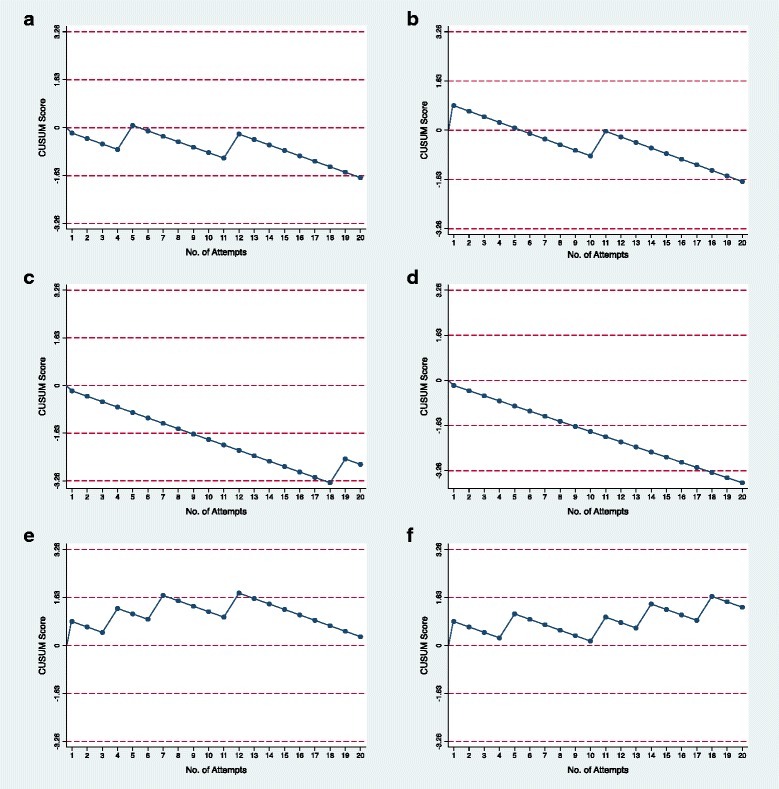
The learning curves were constructed for six anesthesiologist trainees using CUSUM method. **a** total of 20 ultrasound scans of the neck were performed by each trainee. Four out of six trainees. **a**-**d** achieved competency and two trainees did not reach competency. **e**, **f** Each downward point represents a correct identification of the cricothyroid membrane. Each upward point represents an incorrect identification of the cricothyroid membrane. Competency is defined as when the CUSUM score reaches two lines (*dashed horizontal line*) in the downward direction. Failure to reach competency is defined as when the CUSUM score reaches two lines in the *upward* direction

Each trainee performed 20 consecutive neck ultrasound scans for a total of 120 assessments, while a learning curve was built for each trainee. Four out of the six trainees achieved competence in identifying the cricothyroid membrane (Fig. 1a-d). The mean [range] success rate among the four trainees who achieved competence was 94.0% [90–100%]. The median [range] number of attempts required to achieve 90% success rates was 14 [9-18]. Trainee E and F did not achieve competence by CUSUM analysis, but obtained a success rate of 80 and 75%, respectively (Fig. 1e, f). The overall (number of attempts) success rate for the entire group was 88.3% (106/120). The mean (SD) time to identify the midpoint of the cricothyroid membrane was 36.9 (9.0) sec.

Three months after training, all trainees were assessed to identify the cricothyroid membrane using ultrasound on five healthy volunteers. The overall (number of attempts) success rate was 86.7% (26/30) with two and four anesthesiologists had a success rate of 100% (5/5) and 80% (4/5), respectively. The mean (SD) time to identify the cricothyroid membrane was 47.7 (16.0) sec.

## Discussion

Our study determined competency using CUSUM analysis of six anesthesia trainees in ultrasound identification of the cricothyroid membrane of 20 healthy adult volunteers. Our findings showed that a median of 14 consecutive ultrasound scans were required to achieve 90% competence with a mean 94.0% success rate in four of the six trainees. Furthermore, this skill appeared to be maintained 3 months after training.

The total time of the didactic teaching and interactive session was 120 min, suggesting the training required to identify neck landmarks and the cricothyroid membrane with ultrasonography was reasonably short. Furthermore, competence can be achieved with less than 20 scans (range 9–18) and the mean time to perform the ultrasound scan was less than 60 s. These findings imply that ultrasound of neck landmarks and identification of the cricothyroid membrane can be easily learned. In addition, it suggests that pre-procedural use of the ultrasound is not likely to cause a substantial delay in proceeding with the planned airway management.

CUSUM analysis showed that four (Fig. 1a-d) out of six trainees became competent, while two trainees did not achieve competency. However, the success rate of ultrasound identification of the cricothyroid membrane by the non-competent trainees was 80 and 75% (Fig. 1e, f), which is higher than the reported success rate ranging from 34 to 72% by palpation only [7–9].

Learning curves using CUSUM have been used to assess several procedural skills in anaesthesia [20, 21]. The CUSUM analysis is an effective mathematical model to define learning curves for technical skills [18, 22]. It has been employed to investigate competence acquisition in anesthesia technical skills, and more recently, to assess competency in ultrasound-guided procedures [20, 22, 23]. deOliveira Filho have described the learning curves of anesthesiologists for basic procedures such as intravenous cannulation, intubation, spinal and epidural anesthesia [18]. In our institution, we have assessed the proficiency in ultrasound assessment of gastric content and spinal anatomy using CUSUM [20, 23]. It represents a practical and useful tool for objectively measuring performance during the learning phase of basic procedures. When using the CUSUM method in the education context, it is important to understand that it is a statistical method that looks at the outcome rather than the process of performing procedural skills [18, 21].

Previous studies have used expert consensus and institutional results to define unacceptable failure rates [17, 18, 21, 22]. In this study, we defined the acceptable and unacceptable failure rates at 10 and 30%, respectively, based on the performance of previous research on ultrasound assessment [20, 23], and also considering the poor performance of 30% accuracy rate for the standard method of palpation to identify the cricothyroid membrane [7–9].

Ultrasound can be an efficient, life-saving tool to improve the success of cricothyrotomy in patients identified as potentially difficult surgical airway. A cadaveric study by our group demonstrated that ultrasound identification of the cricothyroid membrane significantly improved the success of cricothyrotomy and decreased complications [10]. The American Society of Anesthesiologists [24] and Canadian Anesthesiolgists’ Society [12] airway guidelines reinforce the need to assess and to prepare for a difficult surgical airway scenario with a “double set-up airway intervention”, possibly identifying and marking the cricothyroid membrane before airway manipulation, while incorporating ultrasound and an operator familiar with the technique. The Difficult Airway Society guidelines recommend to identify the trachea and the cricothyroid membrane during the preoperative assessment, and training in the use of ultrasound to evaluate the airway if the landmarks are not clear [13]. Our study supports these recommendations in that competence in ultrasound of neck landmarks and identification of the cricothyroid membrane can be achieved with minimal training. However, there is no evidence for the use of ultrasound during an already established ‘can’t intubate-can’t oxygenate’ airway crisis since preparation and use of the ultrasound can delay the emergency surgical airway required to be performed expediently. A study in the pre-hospital setting showed that open surgical cricothyrotomy was successful in all 42 patients requiring a surgical airway [25]. Identifying and pre-marking the cricothyroid membrane with ultrasound prior to airway manipulation could synergize the open technique by limiting the size of the incision and minimizing complications, particularly, in patients with thick neck and difficult neck landmarks.

Our study has several limitations. First, the small sample size of six anesthesia trainees in our study limits generalizability to a larger population, particularly among practicing anesthesiologists. However, the performance of a diverse group of anesthesia trainees (2 year-1, 2 year-2 residents and two fellows) none with experience in airway ultrasound helps to reassure the feasibility of teaching this technique. Studies have shown that the accuracy to palpate the cricothyroid membrane was low and similar amongst junior and senior anesthesia residents, anesthesia fellows, and practicing anesthesiologists [8, 9]. Second, it is possible that some participants may have used ultrasound as part of their airway assessment during their clinical rotation prior to the 3-month period after training, leading to enhanced ultrasound skill that may potentially bias the assessment at the 3-month period. However, neck ultrasound is not routinely used to assess the airway and it is not currently taught as a mandatory skill required for airway management in our department at our institution (University of Toronto). Third, the anesthesia trainees did not perform the neck landmark palpation prior to scanning in order to avoid bias on the ultrasound examination. However, palpation is always performed in a clinical scenario, as it is still the standard technique to identify the cricothyroid membrane in an emergency situation. Although our volunteers presented with a wide range of anatomic characteristics, including moderate and difficult landmarks palpation, it does not completely represent the clinical patient of a difficult surgical airway with head and neck malformation, tumors and airway trauma. None of our volunteers had an impossible neck to palpate due to excessive tissue. However, excess tissue overlying the cricothyroid membrane could improve focus of the ultrasound beam to provide better image resolution [7, 11]. Nicholls demonstrated that excessive neck tissue in obese patients improves visualization of neck landmarks with ultrasonography and the time to identify the cricothyroid membrane was not affected [11]. In our study ultrasound scans were assessed using the longitudinal technique. Kristensen et al. demonstrated the transverse technique was significantly faster than the longitudinal technique to identify the cricothyroid membrane [26]. It is possible that learning curves might be affected by the different techniques. However, the same authors showed the success rate between techniques were similar [26]. Lastly, we also performed the ultrasound scan in a controlled non-stressful environment. We neither created the stressful environment of a difficult airway situation nor assess directly the cricothyrotomy procedure. However, we previously demonstrated that the context of a stressful simulated airway crisis scenario did not affect the cricothyrotomy procedure [27].

## Conclusions

In conclusion, after a short 2-h training session, most anesthesia trainees (*n* = 4/6) achieved competence in ultrasound-identification of the cricothyroid membrane with less than 20 scans in a mean time less than 60 s., and that they remain reasonably competent 3 months later. The learning curve for ultrasound identification of the cricothyroid membrane seems to be short even without prior airway ultrasound experience.

## Appendix: Cumulative sum method (CUSUM)

Parameters for the construction of a CUSUM graph are the acceptable (*p0*) and the unacceptable (*p1*) failure rates and probabilities of type I and II errors (α and β). We set the acceptable standard of 10% failure rate, the unacceptable standard of 30% failure rate, the probability of a type I error (α) of 0.10, and the probability of a type II error (β) of 0.10. From these, two decision limits (*h1* and *h0*) and the variable *s* are calculated. Since α and β are equal, *h1 = h0* and will be referred to as “*h*”. We then drew decision lines at *h*, *2 h*, and *3 h*, as required, parallel to the X-axis. The graphs start at zero (CUSUM score), and for each successful case, the amount *s* is subtracted from the previous CUSUM score. For each failed case, the amount (*1 - s)* is added to the previous CUSUM score. Thus, a negative trend of the CUSUM line indicates success, whereas a positive trend indicates a failure at the case being analyzed. We considered the anesthesiologist competent at the 10% failure rate, with the probability of a type II error = b if the graph passes two decision lines from above. In this way, early failures did not penalize the anesthesiologist for later increases in accuracy.

Calculation of Sample Size (number or cases):

1. Average number of cases with acceptable failure rate: (*h*0(1−α) − α*h*1) / (*s − p*0)
2. Average number of cases with unacceptable failure rate: (*h*1(1−β) − β*h*0) / (*p*1 −*s*)

a = ln [(1 − β) / α]

b = ln [(1 − α) / β]

P = ln (p1/ p0)

Q = ln [(1 − p0) / (1 − p1)]

s = Q / (P + Q)

h0 = −b / (P + Q)

h1 = a / (P + Q)

## Abbreviations

CUSUM: Cumulative sum
